# A Case of Kawasaki Disease Complicated With Cerebral Salt-Wasting Syndrome

**DOI:** 10.3389/fped.2020.00325

**Published:** 2020-07-17

**Authors:** Masanari Oshima, Junji Fukuhara, Takanori Noto, Teppei Noguchi, Masao Murabayashi, Mamoru Ayusawa, Ichiro Morioka

**Affiliations:** ^1^Department of Pediatrics, Numazu City Hospital, Shizuoka, Japan; ^2^Department of Pediatrics and Child Health, Nihon University School of Medicine, Tokyo, Japan

**Keywords:** Kawasaki disease, cerebral salt-wasting syndrome (CSWS), syndrome of inappropriate antidiuretic hormone secretion (SIADH), hyponatremia, fractional excretion of uric acid (FEUA)

## Abstract

We report the case of a 3-years-old boy who developed severe hyponatremia and unconsciousness during an episode of Kawasaki disease (KD). He was diagnosed with cerebral salt-wasting syndrome (CSWS), which has not previously been reported as a complication of KD. He was diagnosed with KD with fever and four clinical signs and received intravenous immunoglobulin (IVIG) on the day after onset. Hyponatremia had been observed, and it worsened after IVIG. At first, syndrome of inappropriate antidiuretic hormone secretion (SIADH) was suspected, but his hyponatremia did not improve by restriction of water intake. The patient's consciousness level decreased along with the worsening hyponatremia. Electroencephalography revealed abnormal electrical discharge concordant with acute encephalopathy. Laboratory data showed hypouricemia with high fractional excretion of uric acid (FEUA), in addition to a negative balance of both Na and water. We diagnosed KD complicated with CSWS. The patient improved promptly with appropriate Na supplementation and water correction.

## Background

Cerebral salt-wasting syndrome (CSWS), a complication associated with central nervous system (CNS) diseases, is known to cause hyponatremia and dehydration due to excessive discharge of Na and free water (1). Hyponatremia is reportedly observed in 29–70 percent of Kawasaki disease (KD) patients (2). The mechanism of hyponatremia is associated with insufficient intake of Na and syndrome of inappropriate antidiuretic hormone secretion (SIADH) (2, 3). To our knowledge, there have been no reports of CSWS as a complication of KD. CSWS is usually induced by CNS disorders like encephalopathy. We report a case of KD complicated with CSWS following encephalopathy.

## Case Report

A 3-years-old boy was brought to our hospital by his parents because of fever, conjunctival congestion, and rash on the body, all of which appeared the previous day. He did not have any pertinent medical or family history.

At the outpatient clinic, his body temperature was high, and bilateral conjunctival congestion, reddened lips, bilateral cervical lymphadenopathy, and erythema all over the body were noted. Even though the duration of the fever was only 2 days, KD was strongly suspected due to the other symptoms present, and he was hospitalized for treatment.

On admission, his height was 96.7 cm (+0.9 *SD*), and his weight was 13.4 kg (−0.1 *SD*). Body temperature was 39.3°C, pulse rate 153/min, and blood pressure 106/64 mmHg. Other principal signs of KD such as erythema of palms and soles or redness at the BCG inoculation site were not observed. Thoracoabdominal physical findings were normal, and capillary refill time was <2 s.

Laboratory findings on days 2, 6, 8, or 9 and 20 are shown in Table 1. Leukocytes were normal, and C-reactive protein (CRP) was slightly elevated at 0.75 mg/dl. There was no abnormality in the coagulation and fibrinolysis system. Hepatic transaminase was normal, but serum Na was decreased to 132 mEq/L. There were no leukocytes in the urine, and rapid antigen tests were negative for group A beta-hemolytic streptococcus and adenovirus. Although white blood cell (WBC) and CRP values were not typical considering the diagnosis of KD, the five typical principal signs were recognized. Furthermore, his brain natriuretic peptide (BNP) elevated to as high as 68.9 pg/ml (normal range <18.4) on day 6, his erythrocyte sedimentation rate (ESR) elevated to 77 mm/h on day 8, and his platelet count rose to 520,000/μl on day 20. These data retrospectively supported the diagnosis of KD.

**Table 1:** Laboratory data on day 2 (admission) and day 6^*^.

**Hematology**	**Day 2**	**Day 6**	**Day 8**	**Day 20**	**Units**
WBC	5,200	7,400	7,500	8,700	/μl
Neutrophil	78.1	81.8	91.0	70.2	%
RBC	5.08	4.24	4.16	4.16	×10^6^/μl
Hb	13.4	11.4	10.9	11.2	g/dl
PLT	284	292	354	527	×10^3^/μl
**Coagulation**	**Day 2**	**Day 6**			**Units**
PT	13.6	ND			s
APTT	40.8	ND			s
FDP	3.5	ND			μg/ml
Fibrinogen	472	ND			mg/dl
D-dimer	1.9	ND			μg/ml
**Biochemistry**	**Day 2**	**Day 6**	**Day 8**	**Day 20**	**Units**
T-bil	0.3	0.5	0.4	0.3	mg/dl
AST	39	45	63	80	U/L
ALT	17	13	13	15	U/L
LDH	281	272	291	346	U/L
CK	67	53	28	21	U/L
BUN	11.2	13.4	4.3	13.3	mg/dl
Cr	0.38	0.26	0.21	0.28	mg/dl
UA	ND	2.9 (day 7)	2.2 (day 9)	1.4	mg/dl
Na	132	123	126	133	mmol/L
K	4.9	4.5	3.7	4.1	mmol/L
Cl	96	90	94	97	mmol/L
TP	7.6	9.2	7.9	7.5	g/dl
Alb	4.5	3.2	3.1	3.8	g/dl
Ferritin	47.6	ND	ND	ND	ng/ml
s-Osm	ND	263	259	ND	mOsm
BNP	ND	68.9	26.0	ND	pg/dl
**Immunology**	**Day 2**	**Day 6**	**Day 8**	**Day 20**	**Units**
ESR	ND	ND	77	49	mm/h
CRP	0.75	0.08	0.12	0.01	mg/dl
IgG	830	4566	ND	ND	mg/dl
**Rapid antigen test**		**Day 2**			
Group A streptococcus		Negative			
Adenovirus		Negative			
**Urine**	**Day 2**	**Day 6**	**Day 9**	**Day 20**	
Color	Clear	Yellow	Yellow	Yellow	
pH	6.5	7.0	6.5	7.0	
Density	1.025	1.026	1.012	1.009	
Protein	–	±	–	–	
Sugar	1+	4+	2+	–	
Occult blood	–	–	–	–	
WBC	–	–	–	±	
Nitrites	–	–	+	–	
Ketone bodies	1+	2+	–	–	
u-Na	ND	140.1	153.2	29.2	
u-osm	ND	641	395	317	

* *The day when Na value was the lowest*.

*ND, no data*.

Chest roentgenogram showed no heart enlargement or pleural effusion. There were no abnormalities in the electrocardiogram. The largest coronary artery inner diameters by echocardiogram were 1.8 mm (*Z* score – 0.96) for the left main trunk, 1.6 mm (*Z* score – 0.36) for the left anterior descending branch, 1.5 mm (*Z* score – 0.09) for the circumflex brunch, and 2.2 mm (*Z* score + 1.25) for the right coronary artery. Aortic and mitral valve regurgitation and pericardial effusion were not observed.

**Figure 1:**
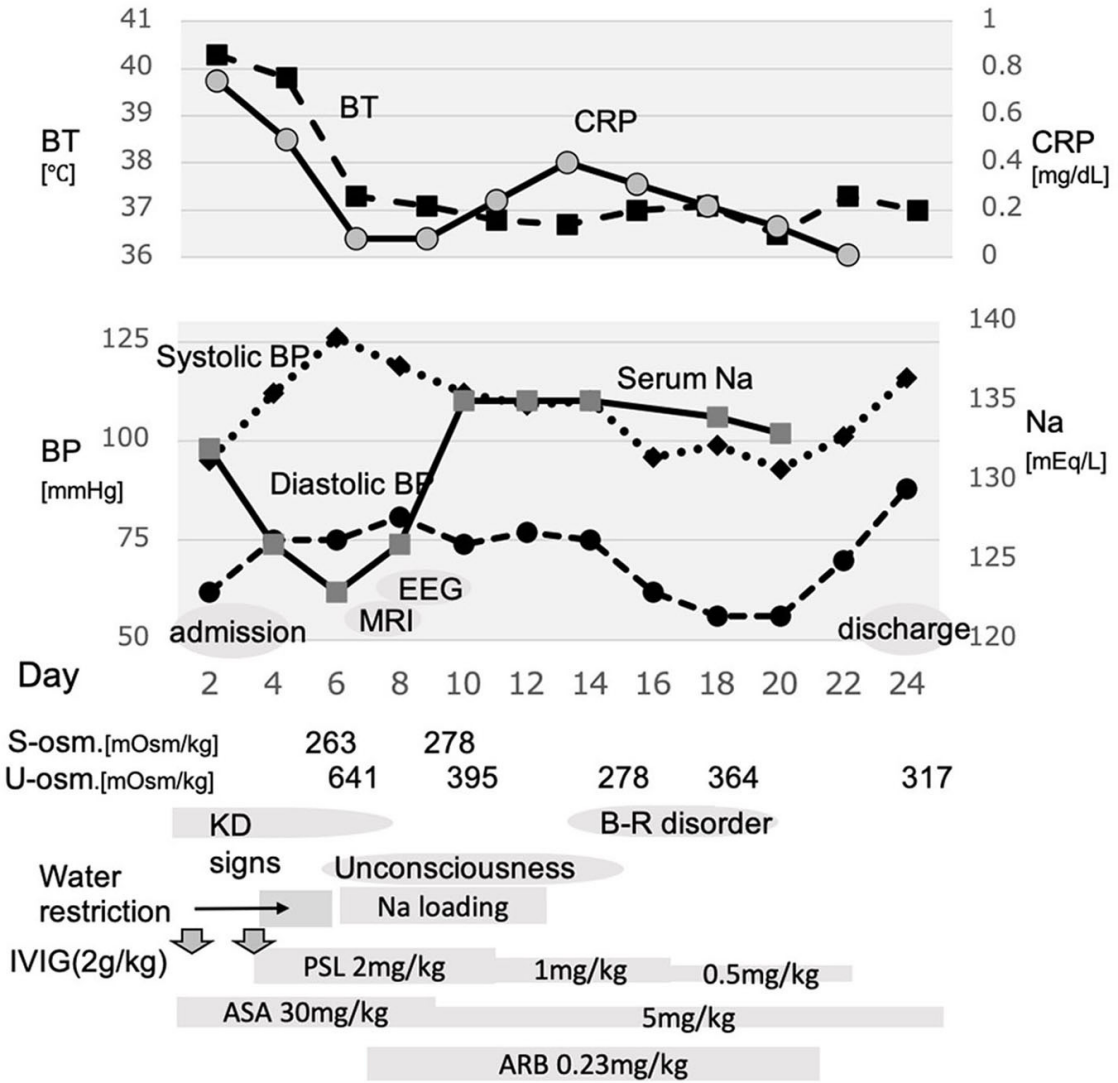
Schema of the course of this case. After admission and treatment with immunoglobulin, moderate fever sustained. Additional immunoglobulin combined with prednisolone was administered, which relieved fever; however, the serum Na level began to decrease, and the consciousness level worsened. Water restriction resulted in worsened hyponatremia. Thereafter, Na loading was started and resulted in improved consciousness and bladder–rectal function. BT, body temperature; BP, blood pressure; s-osm, serum osmolality; u-osm, urine osmolality; EEG, electroencephalogram; MRI, magnetic resonance imaging; KD, Kawasaki disease; B-R disorder, bladder–rectal disorder; IVIG, intravenous immunoglobulin; PSL, prednisolone; ASA, acetylsalicylate; ARB, angiotensin receptor blocker.

Intravenous immunoglobulin (IVIG) treatment and oral aspirin were started on day 2 under the diagnosis of KD (Figure 1). The fever did not resolve, and blood tests on day 4 indicated a high risk of IVIG non-responsiveness on Kobayashi's risk scale (4). A second IVIG was administered on the same day along with prednisolone. As serum Na was 126 mEq/L, water intake was restricted to 700 ml/day as a treatment for hyponatremia potentially due to SIADH. His KD signs including fever resolved gradually on day 5. We continued water restriction. However, as the hyponatremia worsened on day 6 (serum Na 123 mEq/L, Table 1), we considered a possibility of CSWS because urine volume was maintained at no <2 ml/kg/h and urine density was as high as 1.026.

His consciousness was estimated as Glasgow Coma Scale 12 (eye 3, verbal 4, and motor 5). Although dehydration was not clearly evaluated, the intake/output balance of water and Na was negative, urine Na was 140.1 mEq/L, plasma osmotic pressure was 263 mOsm/kg, and urine osmotic pressure was 641 mOsm/kg. In addition, renin–aldosterone did not increase. On day 6, in addition to the correction of water balance, hypertonic saline was started to correct Na.

**Figure 2:**
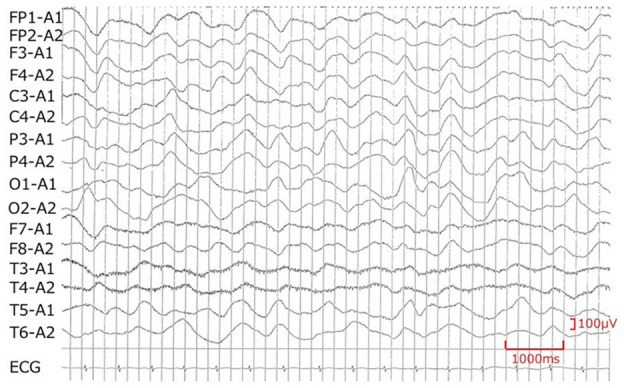
Electroencephalogram recorded on day 8. Encephalopathy is suspected because of the diffuse slowing of the background activity.

Although the brain magnetic resonance imaging (MRI) did not show abnormalities on day 7, the electroencephalogram (EEG) showed diffuse slowing of the background activity, and acute encephalopathy was considered on day 8 (Figure 2). Since the patient's blood pressure had increased after starting prednisolone, oral administration of candesartan was added on day 8. Aspirin was reduced from day 9. Although the unconsciousness was prolonged with mild improvement, hyponatremia improved to 134 mEq/L on day 9. Urine output resumed to >4.5 ml/kg/h after day 9. Prednisolone was reduced from day 11. The patient was troubled with constipation, and an enema was administered on day 14, but he had poor stool discharge. On day 16, as the fecal impaction was remarkable, an enema with water-soluble contrast media (Gastrografin® oral/enema, Bayel Co., Ltd., Osaka, Japan) and manual evacuation were necessary. On day 16, the patient was fully conscious, and on day 20, the patient's urinary catheterization was no longer necessary, and he began to defecate normally. Candesartan and prednisolone were discontinued on day 20. A spinal MRI on day 22 was normal, and he discharged on day 24. No cardiac complications were noted during the course of hospitalization.

## Discussion

CSWS is a syndrome based on CNS disorders in which inappropriate Na loss in urine causes a decrease in fluid volume and hyponatremia. It is known to occur in connection with head surgery, head trauma, or subarachnoid hemorrhage, but in recent years, CSWS associated with meningitis, encephalitis, and encephalopathy have been reported (5, 6). The underlying pathological condition is an increase in Na excretion in the renal tubules, and it is thought that the main site of the pathology is the proximal tubule (1). However, the mechanism by which Na excretion increases has not been clarified yet. It has been suggested that sympathetic nervous system activity involved in Na reabsorption in the proximal tubule and regulation of the renin–angiotensin system and diuretic factors such as atrial natriuretic peptide (ANP) and BNP are involved (7). CNS disorder is always involved in the development of CSWS, and according to the 21st Japanese Nationwide Surveillance of KD, CNS complications such as encephalitis and encephalopathy, including mild encephalopathy with a reversible splenial lesion (MERS), have a reported incidence of 0.09% in KD (8–11).

In our case, consciousness was impaired during treatment of KD, and although the brain MRI was normal, EEG showed generalized slowing of the background activity. Bladder and rectal disturbance was also recognized as a sign of encephalopathy. It is considered that encephalopathy caused suppression of the efferent sympathetic nervous system to the kidney, suppression of the renin–angiotensin system, and loss of Na and uric acid in the proximal tubules together with the decrease in extracellular fluid volume. Furthermore, humoral factors such as BNP may suppress the renin–angiotensin system and antagonize vasopressin in collecting ducts, possibly accelerating the development of CSWS.

When diagnosing CSWS, differentiation from SIADH is essential because distinctly different treatments are required for each condition. CSWS requires water correction and Na loading, while SIADH requires water restriction, and an incorrect diagnosis may worsen either condition. Despite the differences of pathogenesis, with loss of Na and water in CSWS and dilution of serum by water retention in SIADH, both conditions exhibit hyponatremia and have similar laboratory findings. In both conditions, serum osmotic pressure decreases, and urine osmotic pressure increases, while urine Na elevates. Plasma antidiuretic hormone (ADH) levels may also elevate in CSWS; however, it usually takes a few days to get these results. The presence or absence of dehydration and the intake/output balance of Na and water are more useful in differentiating between CSWS and SIADH. Since CSWS patients lose both Na and water, their balances become negative; extracellular fluid decreases, and patients become dehydrated. In SIADH, intake/output balance of Na and water is equal or positive, and extracellular fluid increases due to the increase in ADH.

**Figure 3:**
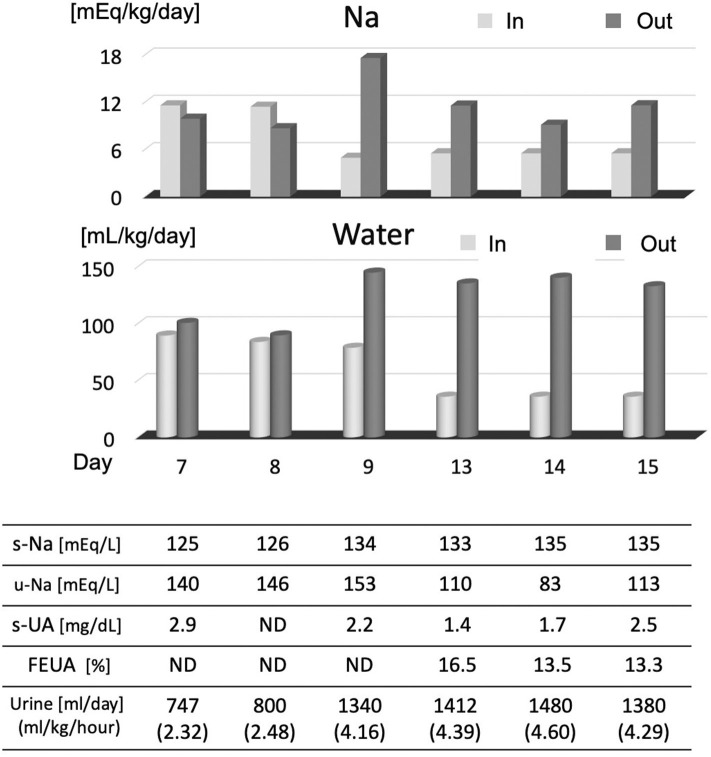
Na and water intake/output balance. After noting the Na loss on day 7, we checked serum Na, uric acid, FEUA, and intake/output balance of Na and water. On days 7 and 8, Na and water intake were increased, and serum Na normalized from day 9. After day 9, the output of Na and water was excessive, and the FEUA increased beyond the normal range (11%), which shows that this condition was CSWS. FEUA = [urinary uric acid (mg/dl) × serum creatinine (mg/dl)]/[serum uric acid (mg/dl) × urinary creatinine (mg/dl)] × 100 (%). UA, uric acid; FEUA, fractional excretion of uric acid; ND, no data.

While the presence or absence of dehydration is an important index for diagnosing CSWS in the early stage of the disease, it is difficult to evaluate the amount of extracellular fluid in children. Measurement of extracellular fluid volume using radioisotope dilution is considered to be effective in terms of accuracy but is not practical in clinical use (12). Also, BUN/Cr, which is generally used as an indicator of dehydration, is not useful in CSWS because BUN does not increase (13). In practice, there is no other method than to evaluate body weight, blood pressure, skin turgor, hematocrit, and inferior vena cava (IVC) diameter. However, in many cases, these measurements are not conclusive unless the dehydration is severe. In our own case, blood pressure and weight were not decreased, and hematocrit was not increased, making it difficult to prove dehydration. However, the balance of Na and water was evaluated from day 7 because of the serious hyponatremia. Both Na and water showed a negative balance, and we diagnosed this situation as CSWS (Figure 3). In this case, urinary Na levels detected in the early stages of the disease were higher than 140 mEq/L, which was more indicative of CSWS than SIADH.

It is reported that changes in serum uric acid level and fractional excretion of uric acid (FEUA) are useful for differential diagnosis (1, 13). The definition of FEUA is “the percentage of urate which was filtered through the glomeruli then was excreted in urine.” It is calculated by the formula shown in Figure 3, and the normal value is below 10–11% (1, 14, 15). Although the mechanism is unclear, serum uric acid levels are low and FEUA are high at the onset of both pathological conditions of SIADH and CSWS. Therefore, CSWS is characterized by continued hypouricemia and elevated FEUA (1). As shown in Figure 3, hypouricemia and elevated FEUA were observed concurrently with hyponatremia in our own case, and these continued after the improvement of hyponatremia, indicating that the condition was CSWS. Furthermore, when hyponatremia was recognized, water was restricted to about 700 ml/day as SIADH was presumed, and the progression of hyponatremia further confirmed the diagnosis of CSWS.

In recent years, there have been sporadic reports of conditions similar to CSWS without CNS disease, and the concept of the disease is changing to renal salt-wasting syndrome (1). Although further elucidation of the pathology is expected, CSWS should be considered when hyponatremia is recognized, even without CNS disease. Though we cannot precisely determine whether CSWS was induced by a neurological complication of KD or by a high-dose infusion of immunoglobulin (3, 16, 17), we should consider various possibilities of low concentration of Na and albumin or impaired renal function. When neurological complications and hyponatremia are present in KD, patients should be assessed for Na intake deficiency, SIADH, and CSWS. Whether or not dehydration is present, care should be taken while assessing the intake/output balance of water and Na, changes in uric acid, and changes in FEUA.

## Data Availability Statement

All datasets presented in this study are included in the article/supplementary material.

## Ethics Statement

Written informed consent was obtained from the minor(s)' legal guardian/next of kin for the publication of any potentially identifiable images or data included in this article.

## Author Contributions

MO, JF, TNot, TNog, and MM were responsible for patient care. MO and MA prepared the draft manuscript. IM gave conceptual advices. All authors critically reviewed and approved the final manuscript.

## Conflict of Interest

The authors declare that the research was conducted in the absence of any commercial or financial relationships that could be construed as a potential conflict of interest.
